# Analytical Approaches to Improve Accuracy in Solving the Protein Topology Problem

**DOI:** 10.3390/molecules23020028

**Published:** 2018-01-23

**Authors:** Kamal Al Nasr, Feras Yousef, Ruba Jebril, Christopher Jones

**Affiliations:** 1 Department of Computer Science, Tennessee State University, Nashville, TN 37209, USA; rjebril@my.tnstate.edu (R.J.); cjone141@my.tnstate.edu (C.J.); 2 Department of Mathematics, The University of Jordan, Amman 11942, Jordan; fyousef@ju.edu.jo

**Keywords:** cryo-electron microscopy, potential energy, protein modeling, protein secondary structure elements, protein topology, geometry, analysis

## Abstract

To take advantage of recent advances in genomics and proteomics it is critical that the three-dimensional physical structure of biological macromolecules be determined. Cryo-Electron Microscopy (cryo-EM) is a promising and improving method for obtaining this data, however resolution is often not sufficient to directly determine the atomic scale structure. Despite this, information for secondary structure locations is detectable. De novo modeling is a computational approach to modeling these macromolecular structures based on cryo-EM derived data. During de novo modeling a mapping between detected secondary structures and the underlying amino acid sequence must be identified. DP-TOSS (**D**ynamic **P**rogramming for determining the **T**opology **O**f **S**econdary **S**tructures) is one tool that attempts to automate the creation of this mapping. By treating the correspondence between the detected structures and the structures predicted from sequence data as a constraint graph problem DP-TOSS achieved good accuracy in its original iteration. In this paper, we propose modifications to the scoring methodology of DP-TOSS to improve its accuracy. Three scoring schemes were applied to DP-TOSS and tested: (i) a skeleton-based scoring function; (ii) a geometry-based analytical function; and (iii) a multi-well potential energy-based function. A test of 25 proteins shows that a combination of these schemes can improve the performance of DP-TOSS to solve the topology determination problem for macromolecule proteins.

## 1. Introduction

To study the relationship between the structure and function of large biological molecular systems, such as proteins, protein inhibitor complexes and macromolecular assemblies, it is crucial to have access to accurate three-dimensional (3D) structural information about the molecule under study. Traditionally, this information has been extracted from the physical item using one of three main biophysical imaging techniques X-ray crystallography, Nuclear Magnetic Resonance (NMR) and, more recently, Cryo-Electron Microscopy (cryo-EM). Unfortunately, to date, the gap between the number of known residue sequences and those with experimentally determined structures is very large. Until relatively recently, X-ray crystallography and NMR have been the dominant experimental techniques used to determine biological macro-molecular structures and have been used to produce the clear majority of such experimentally determined structures. Both suffer from a number of inherent limitations in their utility. Among these limitations are the quantity and quality of sample required for study, the inability to crystalize various molecules and the loss of structural information due to dehydration, crystallization or changes in conformation caused by the removal of in vivo support structures. In the study of relatively small molecules such limitations are troubling, but they become extremely problematic when examining larger macromolecular machines and certain types of proteins, for example, viral capsids, ribosomes, and membrane bound proteins.

A relatively newer method, cryo-EM has proved to be a powerful biophysical technique that is capable of imaging macromolecules in an environment much more similar to their native environment than either X-ray crystallography or NMR can accommodate. In cryo-EM the sample is frozen into a medium and imaged, thus alleviating the need for very pure samples or forced crystallization of the sample. Since less manipulation is required before the molecule is imaged, more of the native structure information is preserved. That is, it does not suffer from the crystallization problem and suffers less loss of native conformal information resulting from dehydration or the removal of membrane support. Cryo-EM is also capable of imaging much larger structures than have traditionally been imaged using X-ray crystallography or NMR. Therefore, it is useful in determining the structure of exactly the sort of molecules that are most difficult to image using conventional methods. These difficult to image molecules are important to medicine and therapeutic treatment of disease. For example, membrane bound proteins account for nearly 50% of contemporary drug targets.

Because of its ability to image these large or membrane supported molecules in relatively impure sample and an environment similar to the in vivo environment, cryo-EM is expected to be the main workhorse in the capture of structural information about the molecular interactions between large complexes within cells [1,2].

For all its promise and potential power, cryo-EM exhibits some drawbacks of its own. It produces volumetric images (we refer to them as volumes in this paper) of the target molecule, generally at sub/nanometer (>5 Å) resolution. Because of the relatively low resolution and volumetric nature of the data, it is challenging to determine atomic scale structural information from cryo-EM volumes. Also, the number of prospective cryo-EM volume on the sub/nanometer scale is rapidly increasing due to the improvement in detectors and other imaging technology. Because of the relative difficulty of analysis of each volume and the need to increase the throughput of analyzed volumes, it is critically important that robust, high performance computational methods be developed to locate atomic scale structures. The development of powerful and automatic computational methods would greatly advance the role of cryo-EM as a complement to traditional diffraction methods.

Computational methods used to model the 3D structure of this class of biological macromolecules (henceforth just called proteins for brevity) can be divided into three main classes: ab initio, comparative, and de novo modeling techniques. In the case in which a target protein is expected to adopt a structure similar to that of a known protein structure comparative modeling can be used [3,4,5]. The existence and identification of a suitable template protein is crucial for this modeling method and finding such a model can be challenging or impossible for some type of proteins, especially membrane bound proteins. If no model can be found, ab initio or de novo modeling can be used.

The ab initio approach attempts to predict the 3D structure of the protein based on its residue sequence. Most ab initio methods combine knowledge-based and physics-based methods to generate the model. The knowledge-based methods allow the prediction of the location of protein secondary structures within the sequence while the physics-based methods are used to determine the potential energy of the proposed model, both are combined to guide the modeling process [6,7,8]. Models are generally scored based on their potential energy, if the potential energy is too high it indicates that the proposed model would be unstable, and that model receives a low score. Due to the complexity of the problem and the vast size of the search space, which increases rapidly as a function of sequence length, ab initio methods are restricted by computational capabilities to relatively small protein molecules.

A third approach, de novo, uses the volumes produced by cryo-EM to model the structure of the protein. Because cryo-EM produces volume files, quantity of the data to be analyzed can be very large. The huge size of the volumes, structural details that require examination and the computational costs of analysis are challenges that must be overcome to use this method effectively. The resolution of the volumes produced by cryo-EM ranges from near-atomic (<5 Å), sub-nanometer (5 Å to 10 Å) to nanometer (>10 Å). At near-atomic resolution, the structure of the molecule can be constructed from the volume. Due to various experimental difficulties, many cryo-EM volumes have been constructed to sub-nanometer (5–10 Å) resolution. At a resolution worse than 5 Å, the volume becomes unclear, and therefore, the atomic model cannot be constructed directly. Computational methods exist that are capable of extracting features from the volume such as the locations and orientations of the secondary structure elements (SSEs) [9,10,11]. 

When a high-resolution atomic structure is available for small proteins or for a part of large proteins, fitting and refinement tools have shown the ability to derive the atomic structure of a protein from cryo-EM volumes [12,13,14,15,16,17]. Given an initial structural model, the volume is used to refine and fit the model structure and to construct a high-resolution all-atom protein model. The refinement process uses a fitting scoring that measures how well the model fits into the volume and identifies mismatched regions between the model and the volume. The techniques that attempt to fit the given atomic structure into the sub/nanometer volumes are called rigid fitting [12,18,19,20,21,22,23,24,25]. In rigid fitting techniques, in which we do not allow changes in the structure being fitted, the goal is to minimize the fitting error with the volume by finding the best corresponding position and orientation. When the atomic structure is not expected to be the same as in the volume rigid fitting cannot be used. To overcome this problem, flexible fitting is used. In this technique, the conformation of the model is modified, in reasonable ways, to improve the fit with the cryo-EM [26,27,28,29,30,31,32,33,34,35,36,37]. In the absence of a high-resolution structure corresponding to a volume, it is not possible to use either rigid or flexible fitting techniques [38]. When this is the case, as it is for many macromolecules, de novo modeling of the protein structure is used.

**Figure 1:**
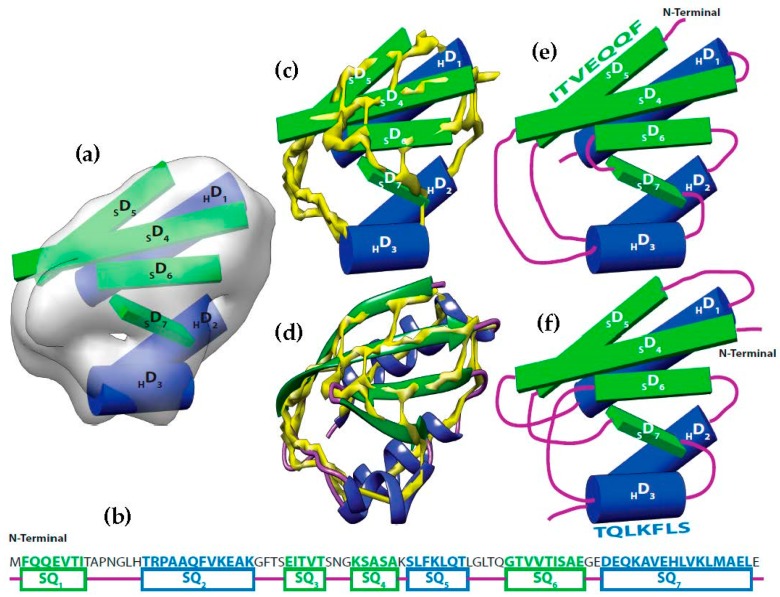
Topology problem. (**a**) The volume and the sticks detected for SSEs-V. The volume was simulated at 10 Å resolution using protein structure 1POH (PDB ID). Three helix sticks (blue) and 4 strand sticks (green) were detected from the volume; (**b**) The SSEs-S observed from protein sequence are marked as SQ_1_ to SQ_7_. Helix segments were colored in blue and β-strands were colored in green; (**c**) The helix sticks were superimposed to the skeleton (yellow) that was generated using the initial version of our skeletonizer [47]; (**d**) The native protein structure was superimposed to the skeleton. (**e**) The correct topology of the SSEs; (**f**) An example of a wrong possible topology.

It is possible to predict, with reasonable accuracy, the location of SSEs, from the cryo-EM volume (SSEs-V). It is also possible to predict, with reasonable accuracy, the location of SSEs from the sequence of the protein (SSEs-S) (see Figure 1). While the prediction from the sequence provides an accurate ordering of elements, the prediction from the volume does not provide any ordering information. This is referred to as the topology problem. Determining this topology is a crucial step in de novo modeling. Most of the de novo approaches that have been proposed solve the topology problem first.

Many different de novo modeling approaches have been proposed [38,39,40,41,42,43,44,45]. Wu et al. [43] used a geometry filter followed by an energetics-based evaluation. Using a pair-wise, knowledge-based approach the energy evaluation calculates potential energies to evaluate for candidates. Because of the computational complexity of these calculations, this method is not suitable for medium or large proteins. Using Gorgon, Baker et al. [39,46] developed a semi-automatic approach to generate the molecular structure. Gorgon [39] solves the topology determination problem first and then, with user intervention, a candidate model of the molecule is built. Since the modeling process requires the involvement of a knowledgeable user the method cannot be completely automated. Further, the system is shown to be insufficient when trying to model large molecules [47,48]. Lindert et al. [38,40] proposed a de novo modeling approach called EM-Fold. Using a Monte Carlo technique, EM-Fold places and orients protein SSEs-S into the cryo-EM volume. An additional Monte Carlo refinement step is then used to improve the number, location, length, orientation and curvature of these SSEs. Following placement of the SSEs, Rosetta’s iterative side-chain repackaging and backbone reconstruction capabilities are used to place side-chains and loops and finally to produce an atomic resolution model [16]. Although this method can work with a large solution space (i.e., up to a sequence length of 500 residues), the stochastic nature of the approach may miss the packing of the protein and thus generate non-native conformations. For a detailed review of structural modeling from EM data, we refer the reader to [49].

Three main approaches have been attempted to address the topology problem of the SSEs [39,40,43,50,51]. Some de novo techniques use the skeleton of the cryo-EM volume in order to reduce the search space and help in modeling [38,40,48]. A skeleton adds another dimension of useful information that highlights the connections between SSEs-V and, therefore, improves the accuracy of finding the correct topology and speed up the modeling process. Al Nasr et al. [52] have formulated the topology determination problem into a constraint graph problem and gave a Depth First Search (DFS) algorithm to enumerate the possible topologies for a protein. Another approach proposed is based on a dynamic programming algorithm for a graph of SSEs, called DP-TOSS, which finds the best-K topologies [48,52]. The experimental results demonstrated the strength of the method with improved accuracy, running time and memory space over existing methods [48]. 

Our approach in this paper is to combine elements of both de novo and ab initio to study the impact on the accuracy of finding the correct topology using DP-TOSS algorithm. We extended the previous work in [53] to include a multi-well potential energy scoring to recast DP-TOSS. We applied three (3) scoring functions individually and as a combination of two (2) or more scoring schemes.

## 2. Results

A set of 25 Cryo-EM volumes and their associated skeletons were used to evaluate the performance of our approach. The volumes of 15 proteins are synthesized to 10 Å resolution using the structure of the protein and the *molmap* command in the Chimera package [54]. A set of 10 experimental volumes and their associated fitted structure are used. Table 1 shows the details of the selected volumes, the fitted proteins, the chain used and resolution of the experimental volumes. The proteins selected for the synthesized and experimental volumes are either helix or β-sheet containing. It is still a challenging problem to detect β-sheet SSEs-V from the sub/nanometer resolution data. Therefore, all SSEs-V were extracted from the native protein structures when aligned with the volumes. The two ends of each SSEs-V were calculated by the center mass of the two end triangles of the first and last three alpha-carbon atoms. To mimic the current challenges of detecting SSEs-V from cryo-EM data, we only extracted the data for helices longer than five amino acids and beta-strands longer than two amino acids. The true location of the helical SSEs-S was generated from the PDB file of the protein structure. Finally, the skeletons were obtained using Skel-EM [47,55].

The topologies were ranked using the six (6) scoring functions listed below Table 2. The correctness evaluation of the two tests was carried out by comparing the produced topologies with the correct topology of each protein obtained from the PDB. The rank of the true topology is then reported in Table 2. A failure is reported (N/A in Table 2) if DP-TOSS could not find the true topology within the top 100 topologies. We used the same skeleton and SSEs-V stick for each scoring test.

Table 2 shows the performance of DP-TOSS before and after incorporating the geometry function, f(φ,η), energy function, $W_{e}$, or a combination of scoring schemes. The test included seven (7) experiments (one not shown) where the DP-TOSS was tested with one recast at a time. For geometry function (Table 2, column 9), the weight of each link $\left(i,j,t\right)\left(i^{\prime },j^{\prime },t^{\prime }\right)$ was updated to f(φ,η) such that φ and η are the dihedral angle and the sum of the two packing angles between SSEs-V j and $j^{\prime }$. For energy function (data not shown), the weight of each link was updated to $W_{e}$. Where $W_{e}$ was obtained from the multi-well potential energy function [56] between the two SSEs-V. A combination of scoring functions was calculated as follow: skeleton (column 5), skeleton + geometry (column 6), skeleton + geometry + energy (column 7), skeleton + energy (column 8), and geometry + energy (column 10). For the skeleton scoring scheme, the weight of each link was scored based only on the traces of the skeleton between the two SSEs-V. Therefore, only $W_{sk}$ was used. For skeleton + geometry scheme, the new weight of each link was $W_{sk}-f\left(\varphi ,\eta \right)$. For skeleton + geometry + energy, the new weight of the link becomes $W_{sk}-f\left(\varphi ,\eta \right)+W_{e}$. Note that the ideal multi-well energy is a negative value. For skeleton + energy, the new weight is $W_{sk}+W_{e}$. Lastly, for geometry + energy scheme, the new weight used was $f\left(\varphi ,\eta \right)+W_{e}$.

## 3. Discussion

In general, the performance of DP-TOSS was not improved in terms of ranking the correct topology within the first 100 for some of the combinations when more than one scoring function is used. Some tests show unchanged performance across some scoring functions (Table 2). For example, tests such as 1FLP (PDB ID) (row 1) and 3HJL (PDB ID) (row 8) have the rank unchanged for some scoring functions (columns 5–8). For most of the tests, the performance was improved noticeably when the skeleton and geometry functions were used. The recast has improved the native (correct) topology by improving its rank to higher positions. For example, the rank of 3ACW (PDB ID) (row 5, column 6) has moved from position 32 to position 7. Similarly, the rank of 4OXW (PDB ID) (row 11, column 6) has moved from position 6 to position 1. Recall that the ultimate goal is to have the native topology for all proteins in rank 1 or near top. More importantly, the geometry function could substantially improve the performance of DP-TOSS when combined with the skeleton traces function for β-sheet containing proteins. For example, the native topologies of the last three proteins were not within top 100 when ranked using the skeleton function in DP-TOSS (column 5). After adding the geometry function, the rank of these topologies has been substantially improved (column 6). This is important when structure evaluation take place. In general, de novo modeling constructs the structural configuration for top-K topologies and further evaluate them using energy calculation. If the native/correct topology is not within the top-K positions, the using of the intensive energy calculation is useless. Thus, our effort to try to guarantee that the correct topology will always be reported within the top ranks.

When a skeleton scoring function used alone (column 5), DP-TOSS was not able to rank the correct topology for some of the β-containing proteins. This is due to the large number of traces, discontinuity and spurs that can be found near the β-sheet regions, which leads to selecting a wrong trace between the SSEs-V and, therefore, the method to fail in ranking the correct topology. This is clear with experimental volumes (rows 16–25). However, we found that the method alone is more stable than all other combinations except for skeleton + geometry (column 6). In general, the ranks produced by skeleton scoring function are within the top 35 for the 68% of the data set and within the top 15 for the 48% of the data set. Generally speaking, the skeleton scoring function is important to stabilize the performance of DP-TOSS. As can be seen from Table 2, DP-TOSS performance degrades when the skeleton scoring is excluded. For example, geometry (column 9) and geometry + energy (column 10) tests show that DP-TOSS performs poorly when the skeleton scoring function is not incorporated. DP-TOSS was able to rank 52% and 44% when geometry and geometry + energy are used, respectively. In addition, only 28% and 16% could be ranked within the top 35, respectively. On the other hand, DP-TOSS could rank 68% of the correct topologies within the top 100 in the worst case when skeleton scoring is incorporated (columns 5–8). Further, in the worst-case test if the skeleton is incorporated (column 8), the correct topologies could be ranked within the top 35 for 40% of the proteins in the data set. This scenario occurs when the multi-well energy is used in the combination.

The multi-well energy scoring function is found to be performing the worst. When used alone (data not shown), DP-TOSS was not able to rank any one of the correct topologies within the top 100. When used with skeleton (column 8) or geometry (column 10), DP-TOSS was able to rank the correct topologies for 68% and 44% of the proteins in the data set, respectively. However, only 40% and 16% of correct topologies were ranked among the top 35. These results show that the multi-well energy function is unstable and cannot be used alone or with only one other scoring function as the main scoring scheme for DP-TOSS. The only case the multi-well energy function performed well is when incorporated with the skeleton and geometry functions (column 7). It was able to rank the 80% of the correct topologies within the top 100. The reason for this poor performance could be because of the way the structures of the SSEs-V were constructed using the ideal dihedral angles and the side-chains were packed. In the original derivation of the multi-well, the authors have used the native structures of the proteins to derive the function. Because of the nature of de novo techniques, the structures are usually built using the ideal dihedral angles. Therefore, the backbone dependent rotamer library and the R3 method used to pack the side chains may choose the wrong side-chains for the amino acids and, therefore, impact the multi-well energy score. More importantly, the multi-well energy function was used to calculate the pairwise energy between SSEs-V not the entire structure.

The same findings are true for the experimental volumes (Table 2, rows 16–25). Although the skeleton and geometry functions (column 6) perform well compared with other combinations, it fails with three proteins (rows 17, 18 and 24). This is expected due to the noise of the experimental volumes. The noise in the experimental data is inevitable. Consequently, more gaps and spurs are expected when the skeleton is extracted. This problem is known for experimental data. Among the 10 experimental volumes, DP-TOSS was able to rank the native topology for seven (7) out of 10 volumes in the best case (column 6) and three (3) out of 10 volumes in the worst case (column 10). On the other hand, DP-TOSS was able to rank the native topology for 15 out of 15 in the best case (column 6) and eight (8) out of 15 for simulated volumes. This shows that experimental volumes are still a challenge for computational tools. This is due to the noise, gaps and uncertainty in the experimental volumes. This challenge appears most clearly when β-sheet is involved. For example, two (2) out of three (3) experimental volumes that skeleton + geometry score fails to rank contains β-sheet (column 6).

## 4. Materials and Methods

### 4.1. Definitions and Notations

At resolution range produced by most cryo-EM volumes, the structure of the protein cannot be derived directly from the volume. Despite this, the spatial description of some features can be discerned using an analysis of density variations. For instance, the location and the orientation of major secondary structure elements in the volume such as helices and β-strands are detectable (Figure 1a) [9,10,11,57,58]. Using different computational methods, the locations of the secondary structures from the sequence of the protein are predictable with accuracy around 80% (Figure 1b) [59,60]. By combining these two items we attempt to automate an early step in de novo modeling. That is, we attempt to find the correct matching (i.e., order and direction) between the SSEs-V and SSEs-S, called topology determination. Topology determination is challenging and has been proven to be NP-Hard [52]. 

Let $SQ_{i},i=1,2,\ldots ,M_{H}+M_{\beta }$ be the segments of amino acid sequence for the helices and β-strands of a protein. Where $M_{H}$ is the number of helices segments and $M_{\beta }$ is the number of strand segments. Due to the linear nature of the protein sequence, the sequence segments have a fixed order $\left(SQ_{1},SQ_{2},\ldots ,SQ_{M_{H}+M_{\beta }}\right)$. Let $\left\{D_{1},D_{2},\ldots ,D_{N_{H}+N_{\beta }}\right\}$ be the set of sticks detected from cryo-EM volume. Where $N_{H}$ is the number of helices sticks and $N_{\beta }$ is the number of strand sticks. In the context of this paper, we assume $M_{x}>N_{x}$, where x is H or β, although vice versa is possible. The topology determination problem can be described as a problem to find a permutation γ of $\left\{1,2,\ldots ,N_{H}+N_{\beta }\right\}$ such that assigning $SQ_{i}$ to $D_{\gamma \left(i\right)},i=1,2,\ldots ,N_{H}+N_{\beta }$ minimizes the assignment score. In the assignment, each $SQ_{i}$ is assigned to $D_{\gamma \left(i\right)}$ in one of the two opposite directions. The total number of possible topologies is $\left(\begin{array}{c} M_{H}\\ N_{H} \end{array}\right)N_{H}!2^{N_{H}}.\left(\begin{array}{c} M_{\beta }\\ N_{\beta } \end{array}\right)N_{H}!2^{N_{\beta }}$. 

The correct mapping of structures detected in the volume and those detected in the sequence must be determined before those items are fitted into the cryo-EM volume. Once the order of SSEs is determined, more conventional techniques can be used model the backbone of the protein and further optimize the structure [38,40,61,62]. Due to factors such as the resolution of the volume and the inaccuracy of detecting SSEs-V and SSEs-S, some de novo methods first find top topologies and then evaluate the resulting models [39,61]. Few approaches have been attempted to address the topology problem directly [39,40,43,50]. One notable approach uses Gorgon [39] and a variant of SSEHunter [10] to find the topology. Another approach uses geometry and energetic-based screening [43]. Recently, a version of a dynamic programming algorithm, called DP-TOSS, which finds the best-K topologies, has been proposed [48,52]. DP-TOSS translates the topology problem into a constraint graph problem and finds the highest scoring topologies using a dynamic programming algorithm. DP-TOSS uses the skeleton of the cryo-EM volume to discover the connections of SSEs-V and, therefore, to find information about the topology of the protein. In this paper, we propose a recast to the scoring function used by DP-TOSS. The recast is based on a geometry and energy analysis carried on protein crystal structures similar to the one used in [43,56]. This updated scoring function is incorporated with the current method used in DP-TOSS to determine the impact on the accuracy of DP-TOSS in determining the topology of protein’s secondary structures.

**Figure 2:**
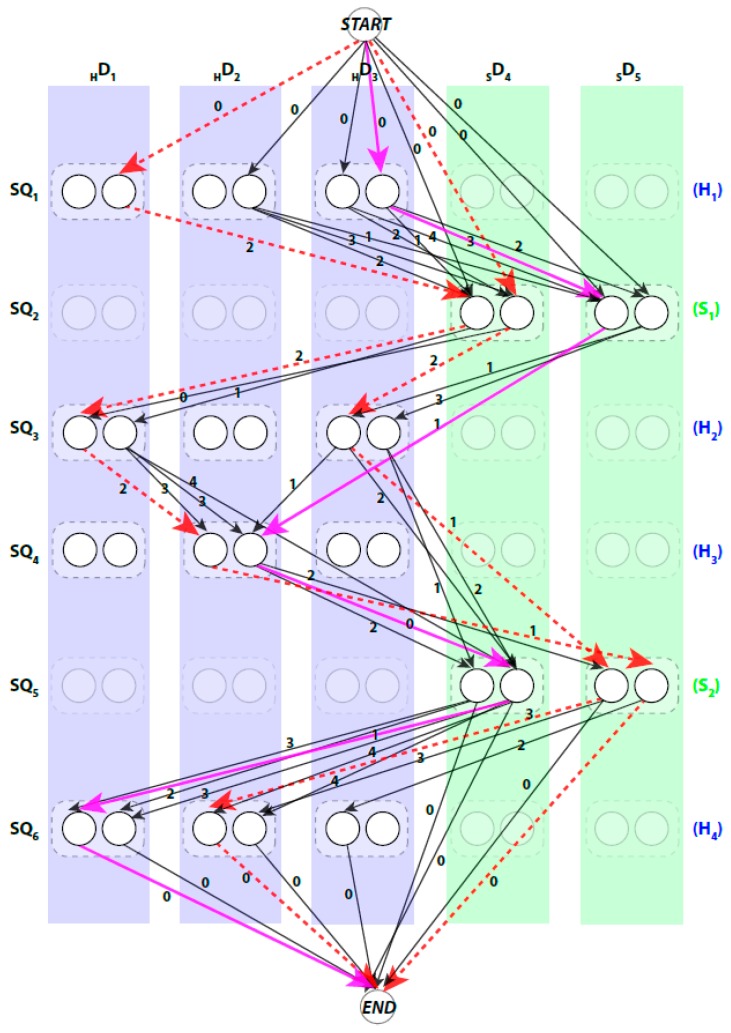
An example of a topology graph. The weights were restricted to integers to save the space in drawing. Two examples of invalid paths are shown in red dashed lines. The shortest path is shown in magenta solid lines. The transparent nodes are nodes that are invalid where the sequence segment is assigned to a stick of different type. Only possible edges are shown.

We use a weighted directed layered-graph $G_{\mathrm{TOP}}$ to address the topology determination problem introduced in DP-TOSS [48]. For details, we refer the reader to [52] and [48]. We briefly outline the main idea of the graph approach here. Let the secondary structure sticks detected from the volume be $\left\{D_{1},D_{2},\ldots ,D_{N}\right\}$, and $N=N_{H}+N_{\beta }$. For convenience, we let $D_{1},D_{2},\ldots ,D_{N_{H}}$ be the helix sticks, and $D_{N_{H}+1},D_{N_{H}+2},\ldots ,D_{N_{H}+N_{\beta }}$ be the β-sticks. Let the set of columns C be {1,2,…,N}. The two ends of a stick are marked by t=0 and t=1 respectively to distinguish the two directions of each assignment. Two nodes are created in each column to represent the two possible directions of the assignment of a sequence segment to a stick. Since a helix segment on the sequence will only be assigned to a helix stick and not a β-stick, V has at most $2M_{H}N_{H}+2M_{\beta }N_{\beta }$ “regular” nodes and two special nodes START and END. The index for the row and column of the nodes is i and j respectively. Each node represents one possible assignment between one SSE-S and one SSE-V in a specific direction. For example, a node (i,j,t) denotes an assignment of sequence segment $SQ_{i}$ to stick $D_{j}$ in t direction and a node $\left(i,j,t^{\prime }\right)$ denotes the same assignment in the other direction (Figure 2). 

The pairwise connection between nodes in $G_{Top}$ can be divided into three (3) types. First, the special connection of the START and END nodes with the other nodes of $G_{Top}$, (START,(i,j,t)) and (i,j,t),END). The weight of these edges is set to zero. Second, the impossible connection, $\left(i,j,t\right)\left(i^{\prime },j^{\prime },t^{\prime }\right)$. This might occur when the Euclidean distance between the two spatial endpoints of the SSEs-V j and $j^{\prime }$, denoted by $vLength\left(j,j^{\prime }\right)$, is longer than the estimated length of the loop between the SSEs-S i and $i^{\prime }$. This means that the structure of the loop between these two SSEs-S cannot fit into the spatial distance between the corresponding SSEs-V. The estimated length of the loop conformation, denoted by $sLength\left(i,i^{\prime }\right)$, is calculated by multiplying the number of amino acids in the loop by 3.8 Å, $sLength\left(i,i^{\prime }\right)=\left(Loop_{\#aa}\left(i,i^{\prime }\right)+1\right)*3.8$, $Loop_{\#aa}$ is number of amino acids between the two SSEs on the sequence. The parameter 3.8 Å is the spatial distance between any two consecutive $C_{a}$ atoms in protein tertiary structures. For example, in Figure 1, to assign the two SSEs-S $SQ_{3}$ and $SQ_{4}$ to any two SSEs-V in any direction, the distance between the endpoints of the sticks for that assignment must be at most 15.2 Å, $vLength\left(j,j^{\prime }\right)\leq 15.2$. When this geometrical constraint is unsatisfied, no edge is corresponding to this connection. The third type of pairwise connection is the possible connection when the Euclidean distance restriction is satisfied between the two endpoints of SSEs-V. The weight of these edges, $\left(i,j,t\right)\left(i^{\prime },j^{\prime },t^{\prime }\right),$ is set to be $W_{Eucl}$, where $W_{Eucl}=sLength\left(i,i^{\prime }\right)-vLength\left(j,j^{\prime }\right)$. Next, we will introduce three different scoring methods for the links of the graph to choose the best one or a combination of more than one approach. 

A path in $G_{TOP}$ begins at START node and ends at END node. When approached in this manner the problem of enumerating the best topologies becomes the problem of enumerating the best paths. Not every path is a valid. For example, those paths that visit the same column more than once are not valid paths, since no stick of the SSEs-V can be assigned to multiple sequence segments. An example of a valid path is shown in magenta thick lines and a non-valid path is shown in red dashed lines (Figure 2). Ideally, the shortest path will represent the true topology of SSEs. However, due to the inaccuracy in the prediction of the SSEs-S and/or detection of the SSEs-V, the true topology is expected to be near the top shortest topologies. DP-TOSS uses a dynamic programming algorithm to find the shortest path and a deviation algorithm based on Yen’s Algorithm [63,64] to find the best-K paths. The running time complexity of the algorithm to find the shortest valid path in the topology graph is $O\left(\Delta^{2}N^{2}2^{N}\right)$, where Δ=M−N+1, M is the number of the SSEs-S, N is the number of the SSEs-V, and M≥N.

### 4.2. Skeleton-Based Scoring

**Figure 3:**
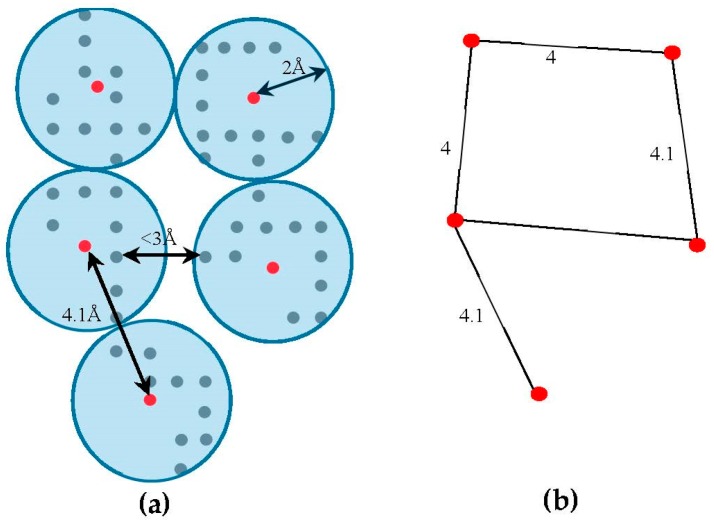
An example of building $SKEL_{G}$ for the skeleton. (**a**) The clusters from REDUCED model are built and centroids (shown in red) are calculated. Each centroid is a node in $SKEL_{G}$ graph; (**b**) Two centroids are connected if the distance between a voxel from the first centroid’s cluster is within 3 Å of any voxel from the second centroid’s cluster. The weights of the edges are the Euclidean distances between the centroids.

The skeleton contains the connection information between the SSEs-V. The length from tracing the skeleton can be used as a strong constraint in matching the SSEs. However, the skeleton often contains gaps and misleading points. In order to estimate the score of the trace connecting the SSEs-V, $W_{trace}$, along the skeleton, we use a graph model. Initially, the regions belonging to the SSEs are removed from the skeleton in order to keep only regions belong to loops. The 3D skeleton is an example of a volumetric image that describes the geometrical shape of the cryo-EM volume. It can be defined on an orthogonal grid, ${\mathbb{Z}}^{3}$. Each point in the skeleton corresponding to a cubic volume called a voxel. The voxel p can be referred to by its orthogonal location (x,y,z). The value saved in the cell corresponding to voxel p represents the associated magnitude of the electron density at that location and is denoted by d(p). The voxels of the grid model can be divided into two classes, foreground and background voxels. If d(p)>0 the voxel is called a foreground voxel. Otherwise, it is called a background voxel. Let SKELETON be the grid cell model of the 3D skeleton. If the voxel p presents in the skeleton, d(p) is set to one and it is called a foreground voxel. Otherwise, it is called a background voxel and its associated density value d(p) is set to zero. Since the input skeleton has many right angles and spurs, we reduce the SKELETON into REDUCED grid model. To build REDUCED, we apply a simple clustering method. Briefly, each cluster is initiated with one random foreground voxel. The size of the cluster is expanded in an iterative method. In each iteration, the method searches for a nearby foreground voxel that is up to 2 Å away from the centroid of the cluster. Every time a voxel is added, the centroid is recalculated. Initially, the centroid is the first foreground voxel that was randomly selected. The process stops, and a new cluster is created, when no more voxels can be added to the current cluster. When no more clusters can be created (i.e., all foreground voxels are assigned to some cluster), the centroids are saved into REDUCED cell model and their density values are set to one. Let $SKEL_{G}=\left(V_{s},E_{s}\right)$ denote the corresponding undirected graph for the centroid voxels in REDUCE, where $V_{s}=\text{\{}v|v=p\;and\;d\left(p\right)=1\}$ is the set of centroid nodes calculated in the clustering step and $E_{s}=\left\{\left(v_{1},v_{2}\right)|Dist\left(v_{i},v_{j}\right)<3.0,v_{i}\neq v_{j},v_{i},v_{j}\in cluster\left(v_{1}\right),v_{j}\in cluster\left(v_{2}\right)\right\}$. The weight of the edge $\left(v_{1},v_{2}\right)$ equals to the Euclidean distance between the two corresponding centroid voxels. Figure 3, shows an example of REDUCED model and its corresponding graph $SKEL_{G}$.

To cut the time required to find the $W_{trace}$ between a pair of endpoints of two SSEs-V j and $j^{\prime }$, we reduce the size of the $SKEL_{G}$ graph. Bron-Kerbosch algorithm [65] is used to find the cliques of size three (3) or more. The purpose of finding the cliques is to find the crowded regions on the graph. The set of nodes involved in the clique are replaced with one central node C, the geometrical central of all voxels of the clique. The edges of the graph will be updated accordingly and the old nodes will be removed. In general, an edge ($C_{i},C_{j}$) will be created if an edge $\left(v_{i},v_{j}\right)\in E_{s}$, where $v_{i}\in C_{i}$ and $v_{j}\in C_{j}$. Due to the noise in the cryo-EM volumes, the skeleton has some gaps and spurs. In order to eliminate the negative impact of this problem, our method tolerates the gaps in the skeleton. To do so, new edges will be added to $E_{s}$ between any two end nodes that are at most 10 Å apart. The end nodes in the graph actually represent the start/end points of the gaps in the skeleton, any node with one adjacent node. Finally, the correspondent nodes in $SKEL_{G}$ to the endpoints of SSEs-V are marked. These nodes are the nodes that located closest to any of the SSEs-V sticks. For each stick, two nodes will be marked as endpoints.

**Figure 4:**
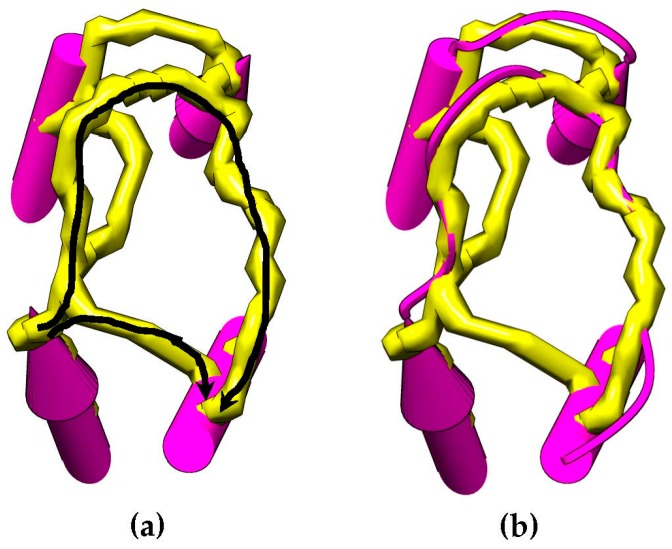
An example of paths for skeleton traces that can be found between two SSEs-V endpoints (**a**). Two paths are found (colored in black arrows). The native loop structure is shown (**b**) that shows that the longest path is the correct path. The length of the two paths is compared with the sLength of the loop and the path that best fits the loop is chosen to calculate the weight of the edge in $G_{TOP}$.

Let $path_{j,t}^{j^{\prime },t^{\prime }}$ be the path between the endpoints of SSEs-V j and $j^{\prime }$ in directions t and $t^{\prime }$, respectively. Let $PATH_{j,t}^{j^{\prime },t^{\prime }}$ denote the set of all paths between the same two endpoints. The cost of a path is simply the summation of edges weights along the path. A depth first search (DFS) can be used to find the paths between a pair of endpoints of two SSEs-V. $W_{trace}$ for the edge $\left(i,j,t\right)\left(i^{\prime },j^{\prime },t^{\prime }\right)$ is calculated by finding the trace on the skeleton that ideally fits the corresponding loop conformation. Thus, $W_{trace}=min\left|PATH_{j,t}^{j^{\prime },t^{\prime }}-sLength\left(i,i^{\prime }\right)\right|$. If a path cannot be found, $W_{trace}$ is set to ∞. Figure 4 shows an example of how the skeleton can be used as an evidence of a connection between SSEs-V sticks. This method is used to change the weighting scheme of the dynamic programing algorithm and improve the accuracy of the topologies to be generated for a given protein. The new weight of the edge, $\left(i,j,t\right)\left(i^{\prime },j^{\prime },t^{\prime }\right)$ is set to be $MIN\left(W_{Eucl}+e,W_{trace}\right)$. Where e is an error constant used to penalize the missing of a skeleton trace between the two endpoints. 

### 4.3. Geometry Analysis

**Figure 5:**
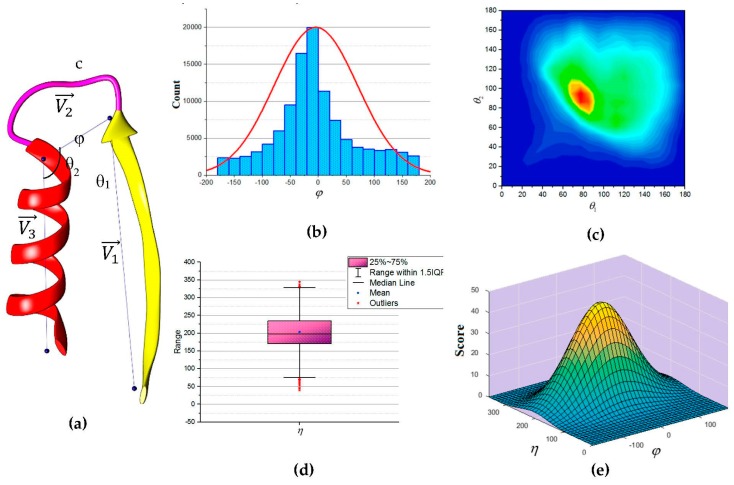
The geometry of consecutive secondary structures. (**a**) The three vectors describe the geometry of the secondary structures and the loop; (**b**) Histogram for the dihedral angle (φ). The curve is the normal distribution with a peak at zero; (**c**) Two-dimensional contour representation of the distribution of angles *θ*_1_ and *θ*_2_. The ridge is along the diagonal line; (**d**) Box plot of the sum of the two packing angles (η ). The box plot is clearly symmetrical overall. The quartiles Q1 and Q3 approximately the same distance from the median. The “whiskers” of the plot approximately the same length; (**e**) Schematic representation of the scaled bivariate normal distribution of the dihedral angle (φ ) and the sum of two packing angles (η ).

In this paper, we propose an update to the current scoring function used in DP-TOSS. The revised scoring function is based on an analysis performed on a total of 110,120 loop structures extracted from a database of 4006 protein structures obtained from protein database (PDB) based on cullpdb_pc20_res2.0_R0.25 PISCES’s list [66]. Three vectors were defined as shown in Figure 5a to describe the packing of a loop and its neighboring SSEs. Two vectors to describe the geometry of the SSEs and one vector to describe the geometry of the connecting loop, $V_{1}$, $V_{3}$ and $V_{2}$ respectively. For helices, the vector defined by the two points calculated from the center mass of the first and last triangle that is calculated by the backbone atoms of the first and last amino acid respectively. The C_α_ atom of the first and last amino acids defines the vector of a β-strand. The end of the first SSE and the start of the second SSE define the loop vector, $V_{2}$. Any packing that is missing any of the atom’s coordinate involved in calculating any one of the vectors was excluded. Two packing angles were defined, dihedral angle φ and packing angles η such that $\eta =\theta_{1}+\theta_{2}$. Inspection of skewness and Kolmogorov-Smirnov statistics indicated that our data were approximately normally distributed for the dihedral angle φ and the sum of the two packing angles η, with a skewness of (0.282) and a kurtosis of (0.098) for φ and a skewness of (0.206) and a kurtosis of (0.082) for η [67]. The histogram for the dihedral angle φ and the box plot for the two packing angles η suggested normality was reasonable, see Figure 5b,d. Hence, the independent angles φ and η exhibit a bivariate normal distribution. 

Thus, we define the scoring function using matrix notation as:

$$f\left(\varphi ,\eta \right)=A\;exp\left(\frac{-1}{2}{\left(x-\Lambda \right)}^{T}\Sigma^{-1}\left(x-\Lambda \right)\right)$$

where A is a normalization scaling factor,

$$x=\left(\begin{array}{c} \varphi \\ \eta \end{array}\right),\Lambda =\left(\begin{array}{c} \Lambda_{1}\\ \Lambda_{2} \end{array}\right)\;and\;\Sigma =\left(\begin{array}{c} \begin{array}{cc} \Sigma_{11} & \Sigma_{12} \end{array}\\ \begin{array}{cc} \Sigma_{21} & \Sigma_{22} \end{array} \end{array}\right)$$

The analytical scoring function, f(φ,η), can be evaluated continuously against dihedral angle φ and packing angles η. In our scoring function there were six parameters $\left(\Lambda_{1},\Lambda_{2},\Sigma_{11},\Sigma_{12},\Sigma_{21},\;and\;\Sigma_{22}\right)$ which were determined to be (−4.501, 203.207, 5581.972, 0, 0, and 2103.773). These values of the parameters were used in the score calculation for each accessible topology candidate.

The links in the topology graph in DP-TOSS are changed based on the analysis. The weight of a link in DP-TOSS evaluates how likely two SSEs-S are to be assigned to two SSEs-V. The new score proposed for a link is: $w\left(\left(i,j,t\right),\left(i^{\prime },j^{\prime },t^{\prime }\right)\right)=f\left(\varphi ,\eta \right)$.

### 4.4. Energy Analysis

The third possible update proposed for the DP-TOSS is the scoring based on the contact energy analysis carried out by [56]. The multi-well potential energy analysis tries to answer the question of the protein topology using the interactions between the secondary structure elements of the protein as the main building blocks. The study has found that the native (i.e., correct) topology is within the top 25% of the ranked topologies based on energy calculations. Although the approach was successful at scoring the native topology within the top portion of the list, the number of possible topologies is tremendously large; therefore, the list of the top 25% is huge. Consequently, the number of topologies to be further analyzed is correspondingly huge. However, the advantage of the method is the ability to include only the secondary structure elements instead of entire protein structure.

The multi-well energy function calculates two terms, the inter- and intra-energy. The inter-energy is calculated based on the interactions between the amino acids from two different SSEs. The intra-energy is calculated based on the interaction between the amino acids within the same SSE. The method first finds the set of amino acids in contact. Two amino acids are said to be in contact if they are within a distance of each other by a given cutoff distance from the center of their side-chain block. The cutoff distance is not fixed. It depends on the type of the two amino acids in question. The center of the side-chain is calculated based on the heavy atoms in the side chain (i.e., C, N and O) and the radius of gyration.

The multi-well potential energy is a modification of the single-well Lennard-Jones [68] function using a set of Gaussian functions. The Gaussian function was used to construct the multi-peak distribution for the interaction between side-chains of all the types of amino acids. For more details, we refer the reader to [56].

To apply the multi-well energy to DP-TOSS, we built the structure of SSEs-V for both types of sticks, helices and β-strands. A general bent backbone structure is first derived. The general values of torsion angles were used to build each type of secondary structure. The FBCCD Algorithm [69,70] was used to bend the structure of the backbone to follow the spline (i.e., central axis). Recall, each SSE-V stick can be assigned to the same SSE-S in two different directions. Therefore, for a given stick, we built two backbone structures, one structure for each node in the $G_{TOP}$ graph. Finally, we added the side-chains of the amino acids using R3 [71] method and back-bone dependent rotamers [72]. The list of amino acids assigned to each stick is based on the node in the graph for that stick. For example, for the node (i,j,t), the sequence of amino acids in segment i is assigned to the stick j in t direction. For example, for the $G_{TOP}$ graph for Figure 2, to calculate the score for the link connecting nodes (3,6,0) and (4,7,1), the nodes assigning the $SQ_{3}$ strand to stick 6 in forward direction and $SQ_{4}$ strand to stick 7 in backward direction, the structures are first derived and the side-chains are packed. Then, the multi-well potential energy is calculated between the two structures and the rest of the portion of the protein are ignored and not involved in the calculation. 

After building the structures for all the sticks/nodes, the approach updates the weight of the edges accordingly. The energy score, $W_{e}$, is the sum of the multi-well inter- and intra-energy between the two nodes/sticks connected by the edge. The new weight of the edge, $\left(i,j,t\right)\left(i^{\prime },j^{\prime },t^{\prime }\right),$ is set to be $W_{e}$ if the two secondary structures are in contact or e, otherwise. Where e is a constant used when no contact energy is found between the two secondary structures.

## 5. Conclusions

Cryo-EM has recently become a major structure determination technique for macromolecule complexes. Cryo-EM produces more volume data every year. Most of the data is not clear enough to visualize the backbone of the protein molecule. Some features such as secondary structure information can be computationally processed from sub/nanometer resolution. Numerous methods have been developed to model protein structure using cryo-EM data. Most of the methods resolve the topology problem of matching between SSEs-V and SSEs-S. Some of these methods use the skeleton of the cryo-EM volume to reduce the search space and to derive the protein structure. DP-TOSS is a dynamic-programming-based computational algorithm capable of finding the correct topology of large proteins. DP-TOSS uses a scoring function to weight the edges of a layered graph. The current scoring function used in DP-TOSS is based on the traces of the skeleton.

In this paper, we proposed a thorough analysis of possible recasts to DP-TOSS’s scoring function. We have analyzed a large database of protein structures and derived scoring terms that describe the packing of secondary structure elements either geometrically or energetically. The current scoring function of DP-TOSS was updated accordingly. A test of 25 proteins showed that some of the proposed scoring terms have improved the performance of DP-TOSS. The geometry packing of the secondary structure elements, when added to the traces of the skeleton, shows the most improvement. The energy packing of the pairwise secondary structures has shown no evidence of any capability of improving the performance of DP-TOSS, even when combined with other scoring schemes. Therefore, we believe that a more careful analysis of other geometry features would improve the DP-TOSS in terms of accuracy and its capability to work with larger proteins.

## Figures and Tables

**Table 1:** The information of the experimental volumes used.

No	EMD ^a^	ID ^b^	Chain ^c^	Resolution ^d^
1	5030	3FIN	R	6.4
2	2526	4CHV	A	7.0
3	8070	5I1M	V	7.0
4	4176	6F36	M	3.7
5	3888	6EM3	L	4.2
6	2843	4UE4	C	7.0
7	8625	5UZB	A	7.0
8	1733	3C91	K	6.8
9	3761	5O8O	A	6.8
10	4154	5M50	C	5.5

^a^ the EM Databank ID of the experimental cryo-EM volume; ^b^ the PDB ID of the fitted protein molecule; ^c^ the chain used in the experiment; ^d^ the resolution of the experimental image in Angstrom (Å).

**Table 2:** The performance of DP-TOSS with different scoring functions.

No.	ID ^a^	SSEs-S ^b^	SSEs-V ^c^	Rank_sk_^d^	Rank_sk+g_^e^	Rank_sk+g+e_^f^	Rank_sk+e_^g^	Rank_g_^h^	Rank_g+e_^i^
1	1FLP	7	6	1	1	1	1	4	17
2	1NG6	9	7	2	2	1	1	7	15
3	2XB5	13	10	11	2	9	47	91	N/A
4	1BZ4	5	5	1	1	3	56	87	N/A
5	3ACW	17	15	32	7	24	28	73	61
6	1A7D	6	4	12	2	17	19	46	94
7	3ODS	21	16	7	1	34	61	N/A	N/A
8	3HJL	20	20	1	1	1	1	4	16
9	1ICX*	13	11	31	12	45	N/A	N/A	N/A
10	1OZ9*	13	12	2	2	3	4	72	N/A
11	4OXW*	8	7	6	1	2	2	18	77
12	1YD0*	8	7	31	5	22	N/A	N/A	65
13	2Y4Z*	8	8	N/A	14	59	92	N/A	83
14	4YOK*	17	15	N/A	37	N/A	87	N/A	N/A
15	4R9A*	14	10	N/A	27	N/A	N/A	N/A	N/A
16	3FIN*	7	7	1	2	2	5	5	24
17	4CHV*	23	19	N/A	N/A	N/A	N/A	N/A	N/A
18	5I1M	19	12	N/A	N/A	N/A	N/A	N/A	N/A
19	6F36	13	7	2	1	3	19	2	63
20	6EM3*	8	8	27	13	77	N/A	51	N/A
21	4UE4	6	5	1	1	3	14	11	56
22	5UZB*	13	7	20	9	29	77	N/A	N/A
23	3C91*	19	19	N/A	51	87	N/A	N/A	N/A
24	5O8O*	24	22	N/A	N/A	N/A	N/A	N/A	N/A
25	5M50*	9	8	41	31	55	82	N/A	N/A

^a^ The PDB ID of the protein used in the test. β-containing proteins are marked with *; ^b^ total number of secondary structure elements in the sequence; ^c^ total number of secondary structure elements extracted from the cryo-EM volume; ^d^ the rank of the correct topology using skeleton traces scoring function, $W_{sk}$; ^e^ the rank of the correct topology using skeleton and geometry function f(φ,η); ^f^ the rank of the correct topology using skeleton, geometry, and energy, $W_{e}$, functions; ^g^ the rank of the correct topology using skeleton and energy functions.; ^h^ the rank of the correct topology using geometry function.; ^i^ the rank of the correct topology using geometry and energy functions.
